# Hybrid and Mixed Matrix Membranes for Separations from Fermentations

**DOI:** 10.3390/membranes6010017

**Published:** 2016-02-29

**Authors:** Christopher John Davey, David Leak, Darrell Alec Patterson

**Affiliations:** 1 Centre for Sustainable Chemical Technologies, University of Bath, Claverton Down, Bath BA2 7AY, UK; C.J.Davey@bath.ac.uk (C.J.D.); D.J.Leak@bath.ac.uk (D.L.); 2 Bath Process Intensification Laboratory, Department of Chemical Engineering, University of Bath, Claverton Down, Bath BA2 7AY, UK; 3 Department of Biology & Biochemistry, University of Bath, Claverton Down, Bath BA2 7AY, UK; 4 Centre for Advanced Separations Engineering, Department of Chemical Engineering, University of Bath, Claverton Down, Bath BA2 7AY, UK

**Keywords:** fermentation, biofuel, separations, mixed-matrix membranes, zeolites, metal-organic frameworks, pervaporation

## Abstract

Fermentations provide an alternative to fossil fuels for accessing a number of biofuel and chemical products from a variety of renewable and waste substrates. The recovery of these dilute fermentation products from the broth, however, can be incredibly energy intensive as a distillation process is generally involved and creates a barrier to commercialization. Membrane processes can provide a low energy aid/alternative for recovering these dilute fermentation products and reduce production costs. For these types of separations many current polymeric and inorganic membranes suffer from poor selectivity and high cost respectively. This paper reviews work in the production of novel mixed-matrix membranes (MMMs) for fermentative separations and those applicable to these separations. These membranes combine a trade-off of low-cost and processability of polymer membranes with the high selectivity of inorganic membranes. Work within the fields of nanofiltration, reverse osmosis and pervaporation has been discussed. The review shows that MMMs are currently providing some of the most high-performing membranes for these separations, with three areas for improvement identified: Further characterization and optimization of inorganic phase(s), Greater understanding of the compatibility between the polymer and inorganic phase(s), Improved methods for homogeneously dispersing the inorganic phase.

## 1. Introduction

Microbial fermentation can be used to produce various fuel and chemical products from a range of renewable or waste substrates. In the interest of sustainable development, these processes can play a huge role in providing an alternative source of fuels and chemicals to those derived from traditional crude oil sources.

**Figure 1:**
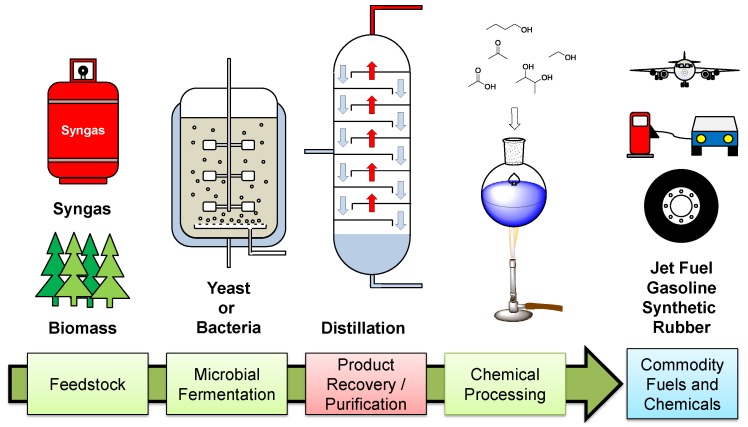
Processes involved in the microbial production of fuels and chemicals.

One of the main disadvantages of the process is the downstream separation of the dilute organics from the fermentation broth (Figure 1). This is an energy-intensive process where the fermentative products are recovered from a complex aqueous mixture of metabolites, proteins, salts, sugars, vitamins and other nutrients used as a growth media for the microorganisms. Distillation is still the dominant separation technology; however, due to the low concentrations of products within the broth it requires the heating of large volumes of water, demanding vast amounts of energy and thereby limiting the cost effectiveness of the process. For example, the recovery of 2,3-butanediol (a precursor to synthetic rubber and jet-fuel) from a fermentation broth, can account for over half the cost of its microbial production [1]. Another example is in the recovery of 1-butanol (a potential alternative road transport fuel) [2] from a 1.5–2.5 wt % acetone-butanol-ethanol (ABE) broth. To recover 1-butanol using a distillation process requires approximately 35–52 MJ·kg^−1^ compared to the energy density of 1-butanol = 36 MJ·kg^−1^ [3]. Other traditional recovery techniques such as chromatography [4] and crystallization [5] have been investigated as suitable recovery technologies for other less volatile fermentation products. These methods have their own limitations: chromatography can be incredibly expensive to scale-up and recovery via crystallization can only be performed on products that will readily crystallize from water. For these microbiologically produced fuels and chemicals to be competitive within the market, they must be cost-comparable with their crude-oil-derived counterparts. Therefore, there has been much research into alternative separation technologies for the recovery of fermentation products. This review aims to give an outline of the role mixed-matrix membranes are playing in achieving lower energy separations of microbiologically produced fuels and chemicals.

## 2. Membrane Processes for Fermentative Separations

Membranes are an important technology providing facile separations for a range of process industries [6]. Membranes act as selective barriers that control the permeation rate of different chemical species. They can retain one solute whilst allowing another to pass freely. There are a number of membrane processes which have been utilized for separations within microbial fermentation and biorefinery processes [7] as they offer substantial savings over conventional thermal separation processes [8]. Examples of microfiltration, ultrafiltration, nanofiltration, reverse osmosis, pervaporation and vapour permeation can all be found in the literature (Table 1).

The majority of commercial membrane processes utilize polymeric membranes due to their flexibility, relatively low cost, and the variety of production processes available. However, polymeric membranes have drawbacks in terms of separation performance due to the flexibility of the polymer chains limiting their ability to discriminate between specific species. The majority of polymer membranes also suffer from various ageing processes over time and can be sensitive to the cleaning agents used in the removal of fouling from the membrane surface [19].

## 3. Inorganic Membrane Materials and Mixed Matrix Membranes

**Figure 2:**
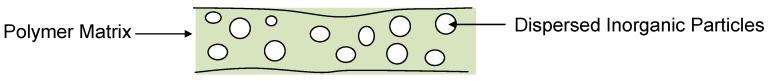
Schematic of a Mixed-Matrix Membrane.

There are a number of different inorganic membrane materials that are currently available commercially or have been investigated within the literature. Although they exhibit remarkable selectivities for a number of significant fermentative separations, there are drawbacks to utilizing inorganic membranes. The membranes are generally brittle and difficult to produce, and are usually produced with thicknesses in excess of 10 μm, resulting in low permeances [20]. Inorganic membranes are therefore very expensive to produce, although there are a variety of novel fabrication techniques being developed to lessen the cost of highly permeable inorganic membranes [21]. To overcome the disadvantages to inorganic membranes, mixed-matrix membranes (MMMs) have been developed. MMMs consist of a dispersed inorganic material within a polymer matrix (Figure 2). MMMs are intended to combine the processability and low cost of polymer membranes with the high separation performance of inorganic membranes. Although these hybrid MMMs generally exhibit enhanced permeabilities and separations compared to purely polymeric membranes, developments must still be made within polymer chemistry to overcome the problems of aging, sensitivity to cleaning agents and fouling. A number of common inorganic materials for incorporation into MMMs within fermentative separations are discussed below; however, examples of many other fillers for MMMs can be found amongst the literature.

### 3.1. Zeolites

**Figure 3:**
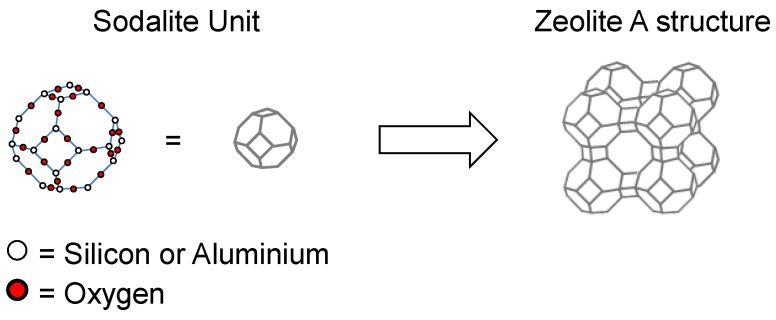
Schematic of a typical zeolite structure made up of sodalite units.

Zeolites are silicalite or aluminosilicate microporous materials (Figure 3). Formed of SiO_4_ and AlO_4_ tetrahedra, they can have a vast number of structures. They are an ideal membrane material due to their uniform, molecularly sized pores as well as their high thermal, mechanical, and chemical stability. Although there are more than 190 zeolite structures reported, only 20 have been shown to give worthwhile separation when prepared as membranes [22] with examples of zeolite membranes present for NF/RO [23,24] and pervaporation processes [25].

In the recovery of fermentation products, zeolites have been studied extensively for their adsorption properties of certain organic products. Hydrophobic and hydrophilic zeolites have been investigated for the recovery of fermentation products. Studies on the adsorption of various fermentation products from aqueous solutions have been conducted on zeolites with high silica ratios due to their increased hydrophobicity. The adsorption of 1-butanol from aqueous solutions has been demonstrated for zeolites with high silica ratios such as silicalite and ZSM-5 and the adsorption studied within the context of the ABE fermentation [26,27,28]. Other fermentation products have also been investigated for adsorptive recovery by zeolites such as acetic acid [29] and lactic acid [30]. Therefore, it is clear how zeolites have become a popular inorganic filler within MMMs for fermentative separation processes due to their ability to separate water from organic solutes.

### 3.2. Metal-Organic Frameworks

**Figure 4:**
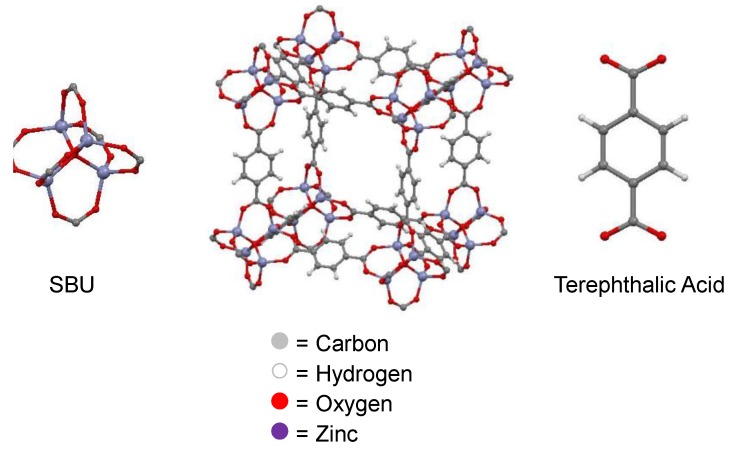
Schematic of the Metal-organic framework (MOF), MOF-5 produced from crystallographic data from [33].

Metal-organic frameworks (MOFs) are a novel type of microporous material (Figure 4). These crystalline materials comprise an organic linker coordinated to a metal or metal oxide cluster (denoted secondary building unit—SBU) [31]. The ability to systematically change the SBU or organic linker allows tailoring of the size and chemical environment of the pores. Widely studied for their gas storage and separation properties, MOFs have received much less attention in their use within liquid separations. For applications within liquid separations, especially those of fermentation broths, MOFs that are chemically stable towards water and organic solvents are required [32]. Although historically MOFs have been highly sensitive to moisture and aggressive media, there are ever increasing examples of robust MOFs that can withstand the conditions required for fermentative separation applications.

With a number of examples of MMMs including MOFs present in the literature for gas separation applications [34], utilization of MOFs within MMMs for liquid and vapour separations (as are needed for separations from fermentations) has received much less attention. Initial investigations have looked into the alcohol/water separation ability of a number of MOFs to ascertain their properties for application in recovery of fermentation products. Initial computational studies have identified a number of MOFs that exhibit suitable water/alcohol separation properties [35,36,37]. For example, De Lima *et al*. investigated the mechanism for alcohol-water separation in the MOF Zn_2_(bdc)_2_(TED) (TED = 1,4-diazabicyclo[2.2.2]octane) [38]. From the density functional theory (DFT) calculations conducted, they deduced that the combination of hydrogen bonding between the alcohol hydroxyl group and oxy group of Zn_2_(bdc)_2_(TED) and the van der Waals interactions between the alcohol alkyl chain with the phenyl ring is the reasoning for the framework exhibiting an increased adsorption of ethanol over water. It is the promise of computational predictions such as this that in part have led to an increasing interest in the use of MOFs for the separation and recovery of fermentation products.

A number of experimental studies have also been conducted on MOFs to identify their water/alcohol adsorption properties. The effect of functional groups on the water adsorption on the MOF MIL-101 was studied [39]. MIL-101 is a MOF that shows high water sorption, and the water sorption profile was successfully manipulated by altering the functionality of the terephthalic acid linker from MIL-101, to MIL-101-NH_2_ to MIL-101-SO_3_H. This ability to alter the sorption behavior of MIL-101 demonstrates the ability to tailor the pore environment of MOFs through synthetic chemistry tools. Water adsorption has also been investigated on a number of other MOFs (HKUST-1, ZIF-8, MIL-101, MIL-100(Fe), DUT-4) with both hydrophilic and hydrophobic materials studied [40].

The adsorption of alcohols on a number of different classes of MOFs has also been investigated [41]. As a sub category of MOFs, zeolitic-imidazolate frameworks (ZIFs) have received much attention recently for biofuel separations due to their high thermal, water and chemical stability [42]. Being composed of tetrahedral metal ions (Zn, Co, *etc*) bridged by an imidazolate ligand, the M-Im-M angle of ZIFs is similar to the Si–O–Si angle (145°) present in zeolites and the synthesis of many ZIFs with zeolite-type tetragonal properties has been achieved [43]. Having high stability towards water and generally exhibiting quite hydrophobic pores, the alcohol and water adsorption within a number of these ZIFs demonstrate great potential for alcohol-water separations [44,45,46,47,48,49]. It has been claimed however, that ZIF-8 is not a suitable membrane material for removal of ethanol from water due to its extremely low initial ethanol uptake, unfavourable diffusion selectivity, and competitive uptake of water [46]. Therefore it is clear why there are a huge number of recent studies including these ZIFs within MMMs.

### 3.3. Carbon Nanotubes

**Figure 5:**
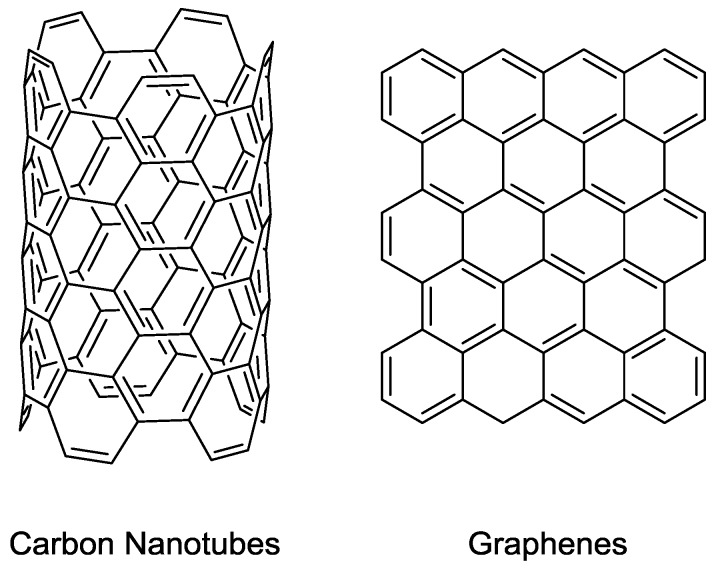
Schematic of carbon nanotubes and graphene.

Carbon nanotubes (CNTs) are a novel form of carbon, which are essentially cylinders of graphene sheets (Figure 5). They have many potential applications in electronics and materials chemistry due to having high strength as well as being highly conducting [50]. Single-walled carbon nanotubes (SWCNTs) consist of a single sheet of graphene rolled into a cylinder and multi-walled carbon nanotubes (MWCNTs) consist of concentric CNTs within each other [50]. It is easy to see how CNTs can be applied toward separation applications due to their uniform channels which can be formed at various sizes and are structurally stable. Therefore, they have found a great number of applications within MMMs [51].

### 3.4. Summary of Inorganic Materials for MMMs

The materials previously discussed above have been some of the main inorganic phases used in MMMs for separations relevant to microbial fermentations. However, there are many more examples of various inorganic materials that have been used for other membrane separation processes but are not discussed herein. The next sections will review the recent literature in the application of these materials in polymer membrane matrices in two of the main membrane processes used for the separation and recovery of organics from fermentations:

- Pressure driven filtrations: ultrafiltration, nanofiltration and reverse osmosis;
- Vapour pressure driven filtrations: hydrophilic and organophilic pervaporation.

## 4. Mixed Matrix Membranes for Ultrafiltration, Nanofiltration and Reverse Osmosis

### 4.1. Overall Process Description and Conventional Membranes

**Figure 6:**
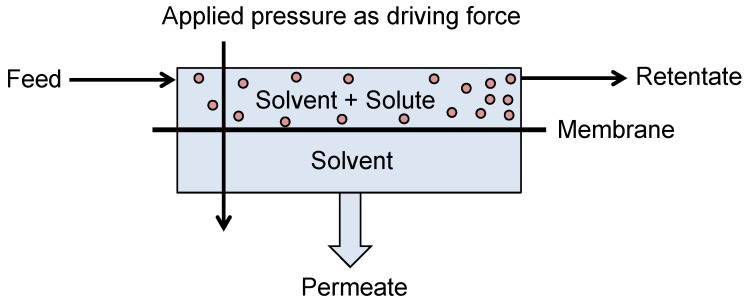
Schematic of a typical nanofiltration and reverse osmosis process.

Nanofiltration and Reverse Osmosis are pressure driven processes which allow for permeation of a solvent and rejection of larger dissolved solutes (Figure 6). NF and RO membranes exhibit MWCOs of between 100 and 2000 g·mol^−1^ and <100 g·mol^−1^ respectively. They can therefore provide the appropriate rejection of many organic solutes within a fermentation media allowing for the purification or concentration of the broth, enabling energy savings within further downstream separation processes.

### 4.2. Polymeric Pressure Driven Membranes

A successful MMM will involve the synergistic combination of a polymer matrix and inorganic filler. Therefore, a brief overview of the common pressure filtration membrane polymers is needed to contextualize the polymers that have been used in MMMs for the same application.

**Figure 7:**
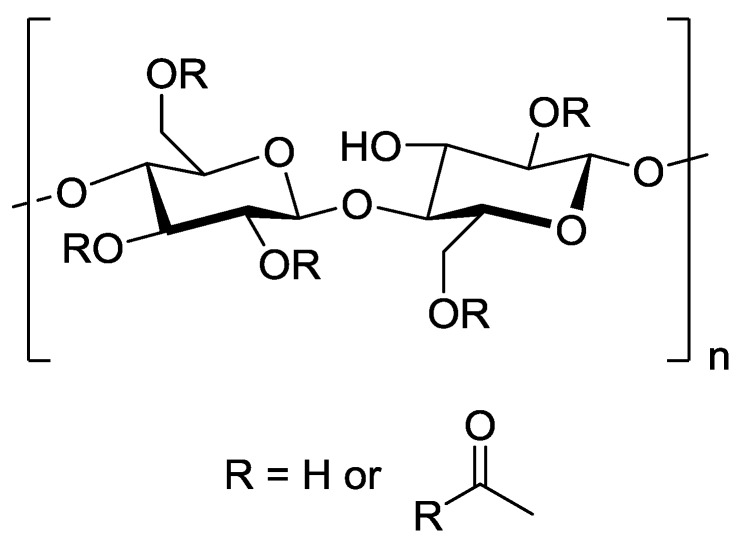
Chemical structure of cellulose acetate.

The majority of initial studies on polymeric RO membranes looked into the use of cellulose acetate (Figure 7) as the barrier layer. Significant studies include those of Reid and Breton [52] who demonstrated that a symmetrical cellulose acetate membrane could retain salt with a 98% rejection, albeit at very low permeate flux, and development of the first asymmetric cellulose acetate membrane gave the first practical demonstration of RO [53]. Since these examples, there has been an extensive range of polymeric materials studied, with various cross-linked polyamides and cross-linked polyetherureas being the most important [54].

**Figure 8:**
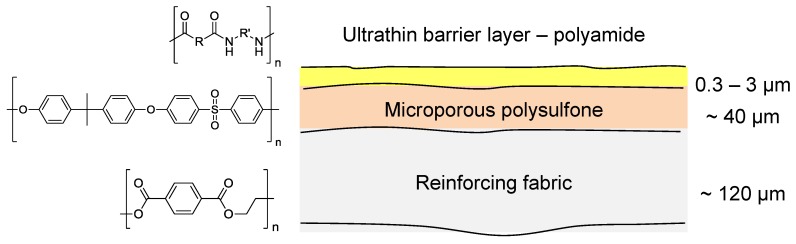
Structure of a typical TFC membrane for NF or RO.

The majority of current commercial polymeric NF and RO membranes consist of a thin-film composite (TFC) structure as shown in Figure 8 [55]. A woven or non-woven support (typically a polyester) is used as a support for a microporous polymer such as polysulfone upon which there is an ultrathin barrier layer of polymer (most commonly polyamide) that controls the rejection characteristics of the membrane.

**Figure 9:**
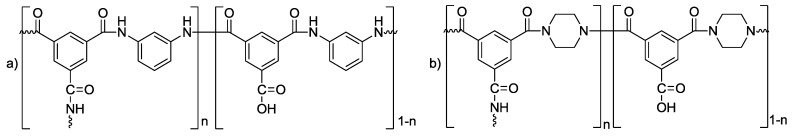
Structures of the active polyamide layer of BW30 (**a**) and NF270 (**b**). The value *n* denotes the amount of cross linking of the polyamide (*n* = 1 when fully cross-linked, *n* = 0 when fully linear).

The structures of current commercial polymeric TFC NF and RO membranes have been studied extensively [56,57]. Figure 9a shows the chemical structure of the fully aromatic polyamide used in many commercial RO membranes, such as BW30. The chemical structure of the semi-aromatic polyamide used in many commercial NF membranes, such as NF270, is presented in Figure 9b. Various modifications to the polyamide molecular structure and the effect this has on the trade-off between salt rejection and water permeability has also been extensively studied [58].

Commercial polymeric NF and RO membranes, such as DDS HC50 [16], NF70 [15], NF270 [59] and DDS HR95 [16], SW30 [59] have been studied for the purification and concentration of various fermentation products. Organic acids such as lactic acid [16,17], acetic acid [13,14] and succinic acid [15] have been purified and/or concentrated using commercial polymeric membranes. NF has been demonstrated to be suitable to remove unused sugars from an ethanol fermentation broth [59] and glycerol has been purified and concentrated from a broth [60]. For the concentration and purification of alcohols and other small solutes there have been fewer studies; for many separations there is still room for improvement in terms of both membrane flux and rejection for commercial polymeric membranes. Traditional polymeric NF and RO membranes also generally exhibit broad MWCO curves [6]. Sharper, more defined MWCO curves would allow for more precise separation of structurally similar compounds and further enable the design of membrane cascades for fermentative separations. This could be achieved through the use of porous inorganic materials with defined pore apertures within the polymer matrix.

### 4.3. Selection of Polymers for Pressure Driven MMMs

Overall, polymers that have already been used in polymer membranes (as detailed in Section 4.2) have also been used for MMMs for pressure driven membrane separations (see Section 4.4 for examples). However, these may not be the most suitable polymers, since the role of the polymer is key in allowing the filler material to have the optimal desired effect. There are many different MMM configurations that are needed depending on the separation mechanism of the polymer and filler desired. For example:

- If the desired effect is for the filler material to have the main selectivity and solute/solvent transport, then the polymer should not present a significant mass transfer resistance and act only as a support. The active surface or pathways of the filler material should be left free (and bypassing of the polymer is fine).
- If both the polymer and filler material impart selectivity and transport of solute/solvent within the separation, then the type of polymer and polymer matrix will be different. The polymer may bind across the active surface of the filler (since bypassing of the polymer is not desired) and the polymer must be defect free.

In all cases, the polymer must allow a tight binding or encapsulation of the filler material to produce stable, robust MMMs. This all indicates that there is significant scope in the future for bespoke polymer development to complement and enhance the different fillers and separations that the resulting MMMs will be applied to.

### 4.4. MOF and Zeolite MMMs for UF, NF and RO

Due to the general aqueous instability of most MOFs, one of the first examples of MOFs within pressure driven membranes was for the creation of macropores within ultrafiltration/nanofiltration membranes, denoted porous matrix membranes (PMM) [61]. The MOFs MIL-53 (Basolite A100), HKUST-1 (Basolite C300) and Fe-BTC (Basolite F300) were included into the polymer matrix of polyacrylonitrile and upon immersion in water left macropores within the membrane structure. This increased permeability whilst retaining a high rejection of dextran. This is a concept that could be applied to ultrafiltration membranes for the clarification of a fermentation broth.

The majority of current commercial polymeric NF and RO membranes are composed of a thin-film composite (TFC) type structure with an ultrathin barrier layer supported on a porous polysulfone support which is further supported on a reinforcing fabric backing layer. The membranes are formed by polymerization of the ultra-thin barrier layer *in situ* over the support material by interfacial polymerization [55]. There are a number of novel advances in NF and RO membrane technology utilizing inorganic materials for water treatment [62]. However, most of these advances have so far not been applied to the separation of fermentation broths, but the new composite mixed matrix membranes have shown promise for a novel way of improving traditional polymeric NF and RO membranes and so will be discussed herein.

**Figure 10:**
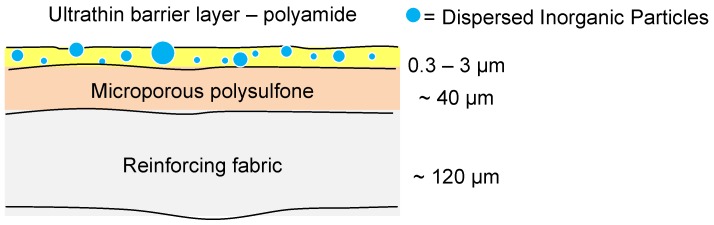
Schematic of a thin film nanocomposite membrane.

As a relatively new concept, thin-film nanocomposite (TFN) membranes include an inorganic filler material within the ultrathin polyamide barrier layer (Figure 10). The inclusion of an inorganic filler within the polyamide layer of a NF or RO membrane allows for potential tailoring of the separation performance of the membranes [63]. The first example of such a composite membrane material by Jeong *et al.* included NaA zeolite nanoparticles in the polyamide barrier layer [64]. The membrane exhibited similar properties to commercial polymeric RO membranes in terms of flux and salt rejection, there have since been many subsequent examples within the literature. Examples have included other zeolites [65], hydrophilized ordered mesoporous carbon (H-OMC) [66], MCM-41 silica nanoparticles [67] and TiO_2_ [68]. Initially TFN MOF membranes were produced for OSNF [69]; however, there have since been many examples of MOFs within TFN membranes for aqueous applications due to the increasing library of water-stable MOFs. ZIF-8 has been shown through molecular simulations to be a suitable inorganic material for an RO membrane [70] and it is therefore clear why it has also been utilized for TFN membranes for water treatment [71,72]. The composite membrane material has been shown to exhibit superior water permeance to a number of commercial polymeric membranes whilst retaining a relatively high apparent salt rejection [71]. Formation procedures and dye removal of a TFN ZIF-8 membrane was investigated by Wang *et al.* [72] who found that having ZIF-8 dispersed in both phases of the interfacial polymerization solutions at a concentration of 0.10% (*w*/*v*) doubled the flux of the membrane and increased dye rejection up to almost 100%. There are still issues, however, with using ZIF-8 as an inorganic filler, as its high adsorption of many organics and water pollutants [73,74,75,76] could decrease a membrane’s rejection of these species, and the long term stability of ZIF-8 in water is questionable [77].

### 4.5. Implications: MMMs for UF, NF and RO

Overall these studies have shown that there has been little work in the use of MMMs to improve flux and selectivity in UF, NF and RO membranes for the separation of fermentation products and so this is a significant area for future research. A wide range of research can be explored in this to-date relatively untouched area.

Therefore there are significant future opportunities in the use of MMMs in pressure driven filtration including:

- The characterization and application of MMMS with a wider range of zeolites and MOFs than currently studied in a wider range of polymer systems.
- The evaluation of a wider range of inorganic filler/secondary phase materials in a wide range of common polymer systems.
- Bespoke polymer development to complement and enhance the different fillers and separations that the resulting MMMs will be applied to.
- The evaluation of mixed filler systems for more finely tuning flux and selectivity to meet the needs of the separation and fermentation products.

## 5. Mixed Matrix Membranes for Pervaporation

### 5.1. Overall Process Description and Conventional Membranes

**Figure 11:**
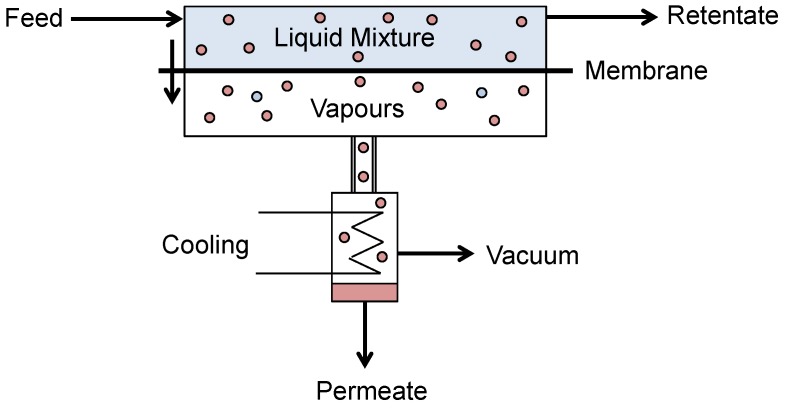
Schematic of a typical pervaporation process.

Pervaporation is a membrane process where a difference in partial vapour pressure is the driving force across the membrane, induced by either a vacuum or sweep gas. Pervaporation combines both evaporation and membrane permeation and is unique among membrane separations in that it involves a liquid-vapour phase change [78]. A liquid feed is contacted with one side of the membrane and vapour is withdrawn from the other (Figure 11).

A pervaporation membrane is defined by the selectivity and flux towards a chosen permeating species. The performance is characterised by the preferentially permeating species, regardless of whether the permeate or retentate is sought. Flux is defined as with all membrane processes and selectivity is generally defined by either the separation factor or enrichment factor:

- Separation factor
  (1) $$\alpha =\frac{A_{P}/\left(1-A_{P}\right)}{A_{F}/\left(1-A_{F}\right)}$$
- Enrichment Factor
  (2) $$\beta =\frac{A_{P}}{A_{F}}$$

where:

▪ *A_P_* = Weight fraction of permeating species A in the permeate
▪ *A_F_* = Weight fraction of permeating species A in the feed
▪ *A_F_* + *A_P_* = 1

**Figure 12:**
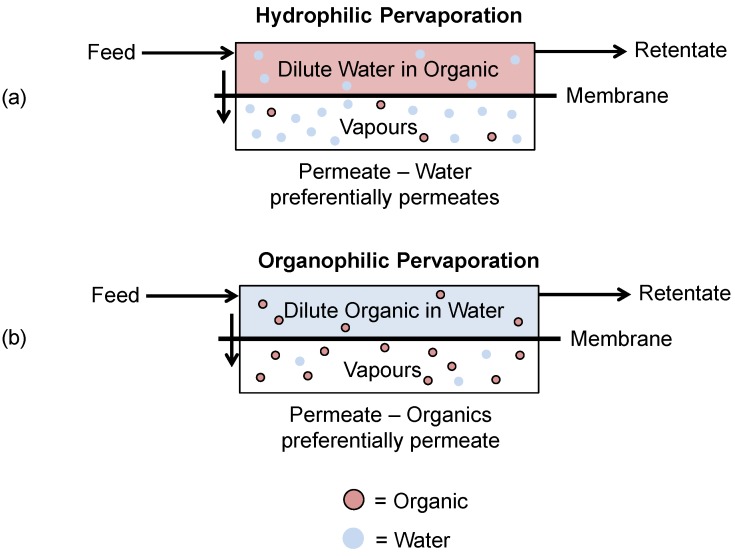
Schematic of (**a**) hydrophilic and (**b**) organophilic pervaporation processes.

Pervaporation for the recovery of fermentation products can be split into two main processes: hydrophilic pervaporation and organophilic pervaporation (Figure 12).

- Hydrophilic pervaporation is used to dehydrate highly concentrated organic solutions through preferentially permeating water across the membrane.
- Organophilic pervaporation utilizes a hydrophobic membrane to recover small quantities of dilute organics from water as the preferentially permeating species across the membrane.

The application of MMMs within these processes for separations applicable to fermentations are discussed in two separate sections below.

### 5.2. Selection of Polymers for Hydrophilic or Hydrophobic Pervaporation Membranes

The selection of the polymer used for a MMM is extremely important to a pervaporation process. The transport across a pervaporation membrane is described as a three-step solution-diffusion-evaporation process and there are a number of transport mechanisms to describe diffusion across a pervaporation membrane [79]. The main factors affecting the permeability and selectivity performance of a membrane will be the solubility and diffusivity through the membrane and the relative volatility of the permeating species [80]. The relative volatility is an intrinsic property of the permeants but the solubility and diffusivity are determined by the membrane material. They are generally considered the rate-limiting steps of the process so long as the concentration of the solutes on the downstream side of the membrane is kept at zero through vaporization using a sufficient vacuum or sweep gas driving force [81]. Therefore as the polymer matrix is almost always the major component of MMMs for pervaporation (in contrast to pressure filtrations, where it may just be a support matrix; see Section 4.3) and so the intrinsic properties of the chosen polymer are hugely important, as well as those of the inorganic filler.

When choosing a polymer for pervaporation it is important to find a polymer that will have a high affinity towards the chosen permeant as well as be stable under the conditions of the pervaporation process. The compaction of polymer membranes by high pressure (which can be problematic in gas separations) is avoided with pervaporation due to the low feed pressure [80]. Other considerations such as cost and ageing of the polymer and having a polymer that has a tight binding or encapsulation of the filler material to produce stable, robust MMMs must be taken into account to produce a membrane that could be successfully applied by Industry.

**Figure 13:**
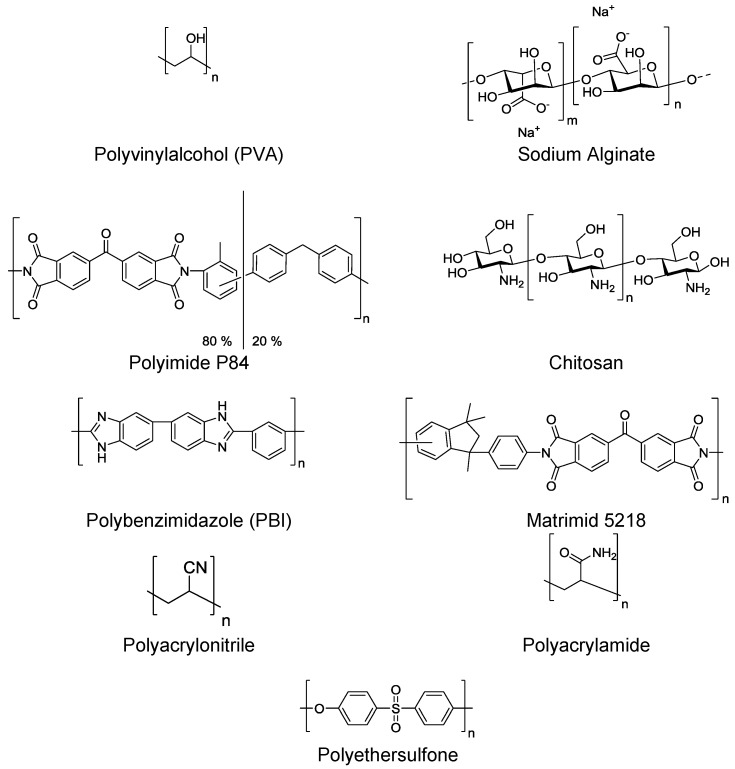
Examples of polymers for hydrophilic pervaporation membranes.

For dehydration of organics via pervaporation, hydrophilic polymers should be used. The hydrophilicity of the polymers arises from the presence of many polar functional groups within the polymer chain (e.g., −OH, −NH, −C=O, −C−O−, −CN) that will interact with water. Examples of these types of polymers used for hydrophilic pervaporation are given in (Figure 13). Polymers which are ionic and neutralized by counterions (such as sodium alginate) are generally water selective as they exhibit high affinities towards it [82].

Table 2 compares a number of polymers tested for the removal of water from concentrated ethanol solutions. As can be seen the choice of polymer used can have a large impact on the performance characteristics of the membrane with the separation factor varying from 12,500 with a polyacrylonitrile membrane down to 52 for a polyethersulfone membrane with flux varying from 0.03 kg·m^−2^·h^−1^ to 0.72 kg·m^−2^·h^−1^ respectively. This demonstrates the importance of choosing the most suitable polymer for the intended separation when fabricating a MMM. Table 2 also presents the difference that the inclusion of an inorganic filler can have on a polymer membrane. For the polyvinylalcohol membrane presented in [83] inclusion of Zeolite NaX at 11 wt % increased the flux to 0.21 kg·m^−2^·h^−1^ but reduced the separation factor to 19.4. This demonstrates the ability to modify a suitable polymer further through the creation of a MMM, which is discussed further in Section 5.3.

**Figure 14:**
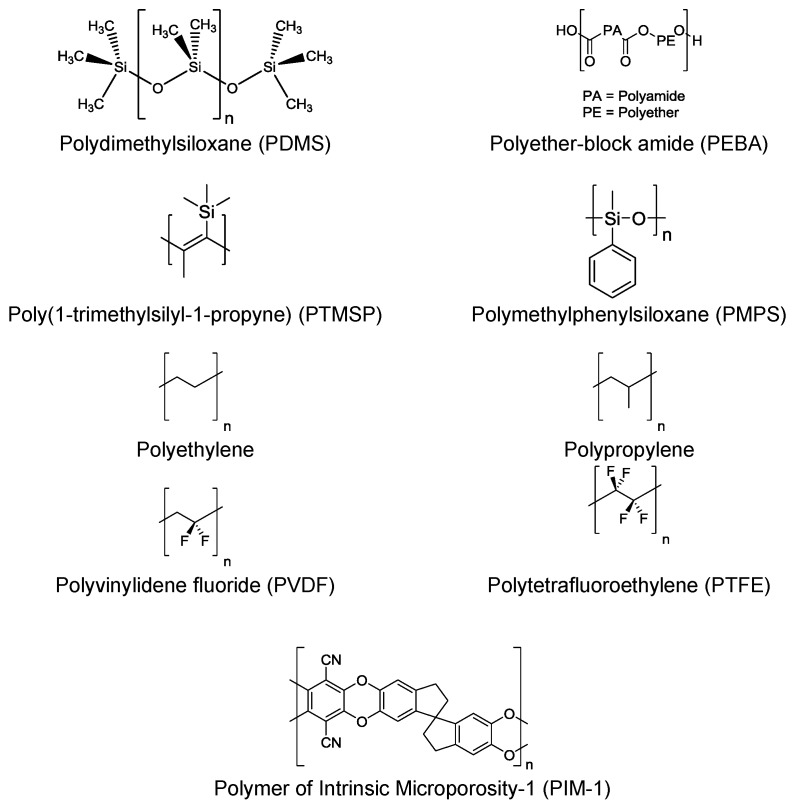
Examples of polymers for organophilic pervaporation membranes.

For organophilic pervaporation hydrophobic polymers are utilized. These polymers possess few or no polar functional groups that exhibit an affinity for water. Examples of polymers utilized for organophilic pervaporation are presented in Figure 14.

Again the choice of polymer can have a significant effect on the performance of a pervaporation process, with a number of studies for organophilic pervaporation by polymer membranes summarized in Table 3. Studies have looked at the effect modifying the functional groups [84,85] of a polymer or through creating new block copolymers [86] have on membrane performance. Again this stresses the need to choose a suitable polymer for the membrane process to be investigated when fabricating MMMs for a specific separation.

Like pressure filtrations, in the main it is polymers that have proven successful for polymer membranes that have been used for MMMs (see Section 4.3). There is therefore a significant opportunity in the future to tailor polymer properties in bespoke polymer development to complement and enhance the different fillers and separations that the resulting MMMs will be applied to.

### 5.3. Hydrophilic Pervaporation MMMs

Pervaporation has successfully been applied for the dehydration of organic solvents. The most successful commercial example being for high purity ethanol for pharmaceutical applications as it can overcome the azeotrope formed with water at ~96 wt % ethanol and it avoids the use of any additional carcinogenic entrainers (e.g., benzene) or a semi-batch (potentially more costly) pressure-swing adsorption [94]. Many other solvents including isopropanol, n-butanol, ethyl acetate, acetone, acetonitrile, pyridine, tetrahydrofuran and n-propanol have also been successfully dehydrated commercially with this technique [81]. Therefore there has been much research into the pervaporative dehydration of various alcohols and other organic solvents [95]. Fermentation products which form azeotropes with water include ethanol at around 4 wt % water, isopropanol at around 12 wt % water and each isomer of butanol (45–12 wt % water). Pervaporation therefore provides an attractive way to dehydrate these fermentation products to >99% purity. Again, MMMs provide an intermediate alternative to costly inorganic membranes and the poorer performing polymeric membranes. Examples of MMMs used for hydrophilic pervaporation are given in Table 4.

#### 5.3.1. Zeolite Filled MMMs for Hydrophilic Pervaporation

Gao *et al.* studied a series of zeolites as potential inorganic fillers dispersed in poly(vinyl alcohol) (PVA) for pervaporative dehydration of alcohols [83]. This systematic study looked at a series of zeolites with increasing pore sizes at 11 wt % in PVA and their separation performance of a number of different sized alcohols to investigate the molecular sieving effect of the zeolites. It was found that the zeolites increased the flux of the membranes due to the increased porosity of the composite; zeolites with larger pore sizes generally increasing the flux the most. The separation factor of small alcohols such as methanol and ethanol, however, was decreased through the addition of zeolites due to their increased permeation through the zeolite pores. However, for larger alcohols the separation factor was increased as well as the flux, mostly for the smaller pore sized zeolites e.g., KA and NaA as the pore sizes are large enough to allow permeation of water but too small for isopropanol or t-butanol to permeate. This therefore demonstrates the importance of understanding the porosity of the inorganic filler and how it will affect the alcohol/water separation to be achieved. Therefore there have been many other investigations into the performance of different zeolites and aluminosilicates within MMMs for dehydration of organic-aqueous mixtures [96,97,98,99,100,101,102,103].

#### 5.3.2. MOF Filled MMMs for Hydrophilic Pervaporation

**Figure 15:**
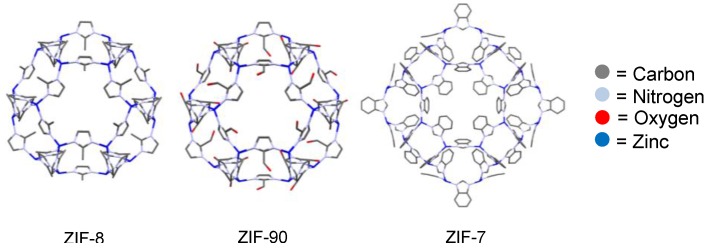
Structures of ZIF-8, ZIF-90 and ZIF-7, hydrogen atoms have been removed for clarity. Structures generated from crystal structure data [42].

MOFs have been investigated for the dehydration of potential fermentation products and examples of ZIF-8, ZIF-90 and ZIF-7 (Figure 15) are discussed.

**ZIF-8** has been used as an inorganic filler in hydrophilic membranes for pervaporative dehydration of alcohols. Examples have included dispersing ZIF-8 in PVA [104], polybenzimidazole (PBI) [105,106] and polyimide (PI) [107]. Although addition of ZIF-8 into the polymer matrix of the hydrophilic polymers increased the flux in each instance, it has generally been observed to be detrimental to the separation factor. For isopropanol dehydration the flux was increased to 992 g·m^−2^·h^−1^ and the separation factor decreased to 91 at 7.5 wt % loading of ZIF-8 in PVA (virgin membrane flux = 135 g·m^−2^·h^−1^ and separation factor = 163). At 58.7 wt % loading in PBI the flux increased to 246 g·m^−2^·h^−1^ whilst the separation factor decreased to 310 (virgin membrane 13 g·m^−2^·h^−1^ and >5000). When the pervaporative dehydration of ethanol was compared for a polyimide membrane with either ZIF-8, mesoporous silica (MCM-41) of two particle sizes (3.1 μm and 0.51 μm) and mesoporous silica coated in ZIF-8, it was expected that because the ZIF-8 aperture size of 0.34 nm is smaller than that of ethanol (0.43 nm) but larger than water (0.27 nm) it would aid the permeation of water [107]. The hydrophobic nature of ZIF-8, however, means it is a poor choice for a hydrophilic membrane as it exhibits no adsorption of water before the onset of capillary condensation [40], whilst also exhibiting high adsorption of alcohols such as ethanol and isopropanol due to the flexibility of the framework [45]. Essentially, by addition of ZIF-8, the membranes have been given a more hydrophobic character, leading to a decrease in separation factor due to increasing the permeance of the alcohols. The ZIF-8 coated mesoporous silica also exhibited poor pervaporation performance within PI due to the ZIF-8 preventing water from entering the silica core. The best performing membrane in the paper was that for inclusion of mesoporous silica of particle size ~0.53 μm, which increased the flux to 440 g·m^−2^·h^−1^ with only a marginal decrease in separation factor to 252 compared to the virgin polyimide membrane (240 g·m^−2^·h^−1^ and 260).

**ZIF-90** on the other hand exhibits a much higher adsorption of water in comparison with isopropanol [45]. Therefore the addition of ZIF-90 into a P84 polyamide membrane was a more obvious choice of filler to enhance both the flux and separation factor of a polymer membrane. Having been included in a P84 polyimide membrane for pervaporative dehydration of isopropanol, the flux of the membrane was increased to 14 g·m^−2^·h^−1^ when compared with the virgin membrane whilst retaining a relatively high water concentration in the permeate (98.4 wt %) [108]. The separation factor was further enhanced by pre-treating the ZIF-90 nanoparticles with sulfonated poly(ether sulfone) (SPES), designed as a primer for nanoparticles, before dispersion in a polymer matrix [109]. The use of SPES to prime the ZIF-90 nanoparticles provides increased particle dispersion by preventing agglomeration of the nanoparticles, improves the affinity between the polymer matrix and inorganic filler and also increases the hydrophilicity of the membrane. The use of SPES as a primer had little effect on the total flux/permeability, but dramatically increased the separation factor of the MMM from 385 to 5668.

**ZIF-7** enhanced the performance within a chitosan membrane for the pervaporative dehydration of ethanol [110]. The performance of the pure chitosan membrane was improved through inclusion of ZIF-7 due to an increased chain rigidification of the polymer which arose from the amino groups of the chitosan interacting with the Zn of the ZIF-7 particles. Although ZIF-7 exhibits the same hydrophobicity and topology as ZIF-8, the framework exhibits a narrower pore aperture (0.30 nm compared to ZIF-8 = 0.34 nm) [42] and a more rigid framework [111], which has been used to explain the much lower adsorption of isobutanol when compared to ZIF-8 [112]. This means that ZIF-7 does not easily allow the permeation of water or ethanol which decreases the flux of the chitosan membrane; however, the rigidification of the polymer means that ethanol does not pass as easily through the membrane whilst smaller water molecules can still freely pass. This resulted in an increase in separation factor for the MMM; at 5 wt % loading of ZIF-7 the chitosan MMM exhibits a flux of 322 g·m^−2^·h^−1^ and separation factor of 2812 when compared to the chitosan membrane; flux = 602, separation factor = 148.

#### 5.3.3. Nanotubes, Carbons, and Other Filled MMMs for Hydrophilic Pervaporation

The use of CNTs for pervaporative dehydration of alcohols has also been investigated. Shirazi *et al.* incorporated CNTs into a PVA membrane for pervaporative dehydration of isopropanol [113]. CNTs are prone to agglomeration due to Van der Waal forces, which means that dispersion within a polymer matrix can be difficult. The acid treatment of the chemical vapour deposition (CVD) produced CNTs was used to purify the CNTs and improve the dispersion. With increased loading of CNTs the degree of swelling by a 20 wt % water IPA mixture was observed. Addition of CNTs decreased the permeation flux of the membrane but increased selectivity from 119 for the pure membrane to 1794 for the membrane up to 2 wt % of CNT. At a 4 wt % loading of CNT flux increased but lowered separation factor due to agglomeration of the CNTs creating nonselective voids within the membrane. Another strategy used to prevent agglomeration of CNTs within a PVA membrane was to wrap the carbon nanotubes in poly (alylamine hydrochloride) (PAH) [114]. PVA membranes were prepared with MWCNTs produced via CVD with and without wrapping with PAH. The selectivity of the membranes for dehydration of an isopropanol/water (90/10 wt %) feed at 30 °C were increased through addition of the MWCNT-PAH and at 1 wt % loading increased the separation factor (141 to 945) with only a marginal decrease in total flux (229 to 207 g·m^−2^·h^−1^) when compared to the virgin PVA membrane. However above 1 wt % loading the MWCNT-PAHs agglomerated and decreased the separation factor of the membrane.

Functionalised graphene sheets (FGS) have been utilized as an inorganic filler for hydrophilic membrane preparation. Firstly incorporated into sodium alginate chitosan membranes the FGS improved the hydrophilicity of the membrane with increased loading and performance of the membrane improved with optimum selectivity at 2 wt % of FGS for isopropanol dehydration [115]. Further work by Dharupaneedi *et al.* prepared FGS-chitosan membranes for ethanol and isopropanol dehydration [116].

Shivanand *et al.* prepared MMMs from a heteropolyacid (HPA) and PVA [117]. Increased loading of the acid dramatically decreased the flux of the membrane whilst dramatically increasing the separation factor of the membrane.

The addition of sodium montmorillonite (Na^+^MMT) clay into PVA membranes for dehydration of organic solvents has also been studied. By incorporating the hydrophilic Na^+^MMT particles into the membrane the hydrophilicity of the membrane was increased increasing the separation factor as well as demonstrating increased mechanical properties of the MMMs. This increased the membrane separation factor from 77 to 2241 for 10 wt % loading of the clay with a flux of 77 g·m^−2^·h^−1^ [118].

**Figure 16:**
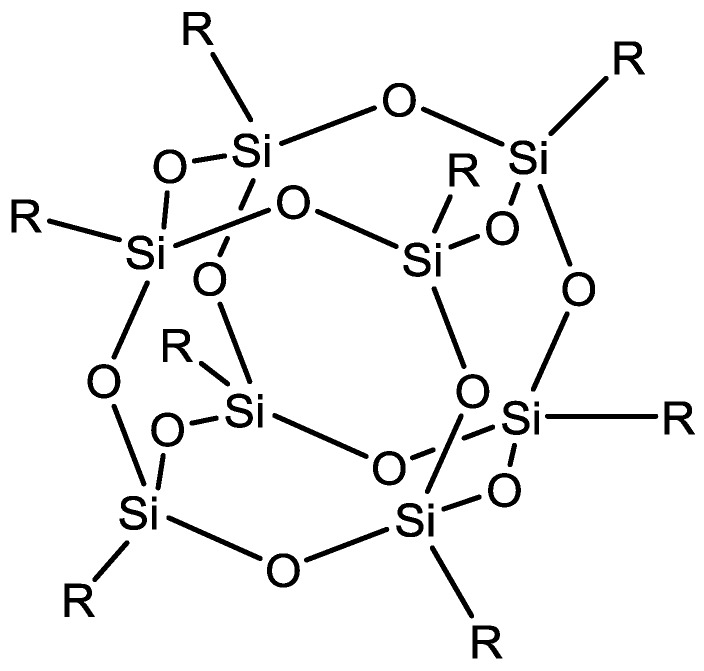
Structure of a typical polyhedral oligomeric silsequioxane.

Another filler used within MMMs for ethanol dehydration is polyhedral oligomeric silsesquioxanes (POSSs) (Figure 16). POSSs are cages of silicon and oxygen with each silicon atom bonded to three oxygens as well as an alkyl, halide, aryl or alkoxy group. POSSs have been embedded within the polymer matrix of copoly (1,5-naphthalene/3,5-benzoic acid-2,2'-bis(3,4-dicarboxyphenyl) hexafluoropropanedimide (6FDA-NDA/DABA) hollow fibres. The separation factor for these hollow fibres for ethanol/water mixtures was 166 and flux total flux of 1900 g·m^−2^·h^−1^ [119]. POSS was also incorporated into a novel polymer blend of the polyimide and sulfonated polyimide material to further improve the performance characteristics of these hollow fibre membranes [120].

#### 5.3.4. Implications: MMMs for Hydrophilic Pervaporation

Overall these studies have shown that inorganic fillers can improve the flux and/or separation factor of hydrophilic pervaporation membranes. It is important, however, to understand the intrinsic properties of the inorganic fillers such as affinity to the feed components to design MMMs with improved performance characteristics.

Future opportunities in the use of MMMs for the hydrophilic pervaporation of potential fermentation products may include:

- Further investigations into the effect of different membrane fabrication techniques on the performance of the described hydrophilic MMMs for applicable separations to fermentations.
- Further screening of hydrophilic MOFs as inorganic fillers.
- Integrating a high-performing MMM into the final purification step in the pervaporative recovery of bioethanol (or similar fermentation product) from a biorefinery and study of the long term operation of such a membrane.

### 5.4. Organophilic Pervaporation

Organophilic pervaporation can recover/remove dilute organics from aqueous solutions. Compared to hydrophilic pervaporation it has found little commercial success despite being intensively researched. The process, however, could provide huge potential energy savings in the recovery of fermentation products. Organophilic pervaporation has generated a lot of interest in the recovery of 1-butanol from the ABE fermentation to increase the production efficiency [125] with a number of polymeric membranes being studied [126]. For organophilic pervaporation to become viable however, the key issues which must be addressed include: improvement of the alcohol–water separation factor, increasing membrane flux and a greater understanding with regards to fouling and module stability [127]. It is hoped that the use of novel inorganic fillers within polymeric pervaporation membranes may be able to alleviate or resolve some of these key issues for pervaporation as a biofuel recovery method. Consequently the development of stable and high performing MMMs is key to the future of organophilic pervaporation. Examples of MMMs used for hydrophobic pervaporation are given in Table 5.

#### 5.4.1. Zeolite Filled MMMs for Organophilic Pervaporation

As discussed in Section 3.1, zeolites have been reported to have separation ability for water/alcohol mixtures. Therefore there are many examples of zeolite MMMs for pervaporation. Being a hydrophobic sorbent that exhibits preferential adsorption of alcohols from aqueous solutions as well as desorption of these alcohols at relatively mild conditions it is obvious why Hennepe *et al.* utilised silicalite-1 as an inorganic filler for a polydimethylsiloxane (PDMS) organophilic pervaporation membrane [128]. The inclusion of silicalite-1 into the PDMS matrix increased the separation factor and flux for pervaporation of methanol, ethanol, 2-propanol and 1-propanol. Hennepe *et al.* also presented how part of the transport through the membrane was through the pores of the silicalite-1 particles. Jia *et al.* prepared a thin film composite membrane of silicalite-1 and PDMS [129]. The membrane exhibited higher fluxes than that of Hennepe as well as a greater separation factor for the pervaporation of ethanol from water. Silicalite-1-PDMS membranes have also been applied to the pervaporation of 1-butanol [130]. To aid application within fermentative separations of 1-butanol, silicalite-1-PDMS membranes have been studied at low concentrations of 1-butanol relevant for in-situ removal of butanol from a fermentation broth [131] and studied in relation to the ABE fermentation process [132,133].

**Figure 17:**
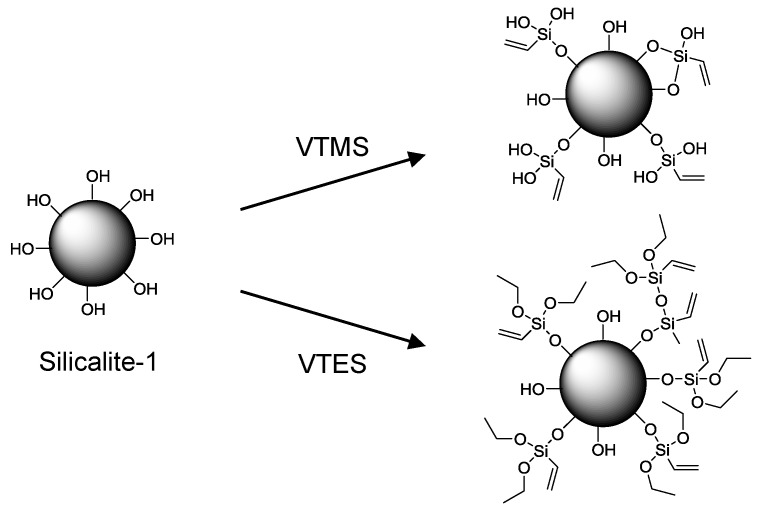
Modification of silicalite-1 with VTES [136] and VTMS [137] showing the incorporation of vinyl groups to the surface.

Various efforts have been made to improve the performance of these membranes by increasing the dispersion and homogeneity of silicalite-1 within PDMS. One approach was to use nanosized silicalite-1 [134] and recently the effect of different sizes of nano silicalite-1 has been investigated [135]. Both studies also investigated the effect of surface modification through silylation of the nano-scale silicalite-1 and this has become a popular method of increasing the compatibility between silicalite-1 and PDMS. Other reports of silylation of silicalite-1 for MMMs of PDMS include modification with vinyltriethoxysilane (VTES) [136] and vinyltrimethoxysilane (VTMS) [137] (Figure 17). For the VTMS modified silicalite-1, the separation factor was improved from 120 to 145 when compared to the unmodified silicalite-1-PDMS membrane. Chlorosilanes have also been used to increase the surface hydrophobicity of silicalite-1 for pervaporation within a PDMS MMM [138].

Investigations have also looked at the inclusion of silicalite-1 within different polymers that have shown high pervaporation performance such as polyether-block amide (PEBA) [139] and polymers of intrinsic microporosity (PIMs). PIMs are novel polymers that utilize inefficient packing of the polymer chains through restricting the rotational freedom of the polymer backbone to induce microporosity [140]. PIMs have been shown to produce organophilic membranes [90,141,142] and the inherent microporosity means that they are an ideal pervaporation membrane material. A Silicalite-1-PIM-1 MMM has been fabricated for the pervaporation of ethanol from dilute aqueous solution [143]. The incorporation of silicalite-1 at high loadings increased the separation factor compared to that of the purely PIM-1 membrane to 5.68 compared to 3.61.

Another zeolite, ZSM-5, has been reported by Vane to enhance the ethanol permeability of a PDMS membrane with increased zeolite loading [144]. After this Vane also reported on the long term operation of these membranes with an S. cerevisiae broth [145]. Exposure to the broth caused a large decline in permeability of the membrane due to the many components of the broth. ZSM-5 has also been incorporated into PEBA for pervaporation of 1-butanol [146].

#### 5.4.2. MOF Filled MMMs for Organophilic Pervaporation

MOFs have gained a huge amount of interest for inclusion within organophilic pervaporation membranes in recent years. The first example of a MOF MMM for organophilic pervaporation looked at two analogous single crystal adsorbents [147]. The vapour adsorbency of methanol, ethanol, 1-propanol, 1-butanol and 1-pentanol were studied for the single crystal adsorbents [M^II^_2_(bza)_4_(pyz)]_n_ (M = Rh or Cu, bza = benzoate, pyz = pyrazine). The copper adsorbent was fabricated into a PDMS membrane and the alcohol water separation for methanol and ethanol studied. The inclusion of the copper complex increased both flux and separation factor in both instances however the membrane performance is well below that for other MMMs.

**ZIF-8** (Figure 15) has been demonstrated to separate alcohols from water due to its hydrophobic pores exhibiting high uptakes of alcohol and low uptakes of water [45,47,48,49]. Therefore it is clear to see why it was originally incorporated into a polymethylphenylsiloxane (PMPS) membrane [112]. MMMs of ZIF-8 and PMPS were produced within alumina capillaries by the solution-blending dip-coating method. The membrane was mainly tested for the pervaporation performance for removal of isobutanol from an aqueous solution due to the high adsorption of isobutanol observed for ZIF-8. The high permeance of isobutanol was attributed to the ZIF-8 particles dispersed in the membrane creating preferential pathways for the permeation of isobutanol even though the pore aperture of ZIF-8 (as calculated from crystal structure data) is smaller than the size of isobutanol. It was suggested that the high adsorption of isobutanol and increased permeance of the membranes was therefore due to the gate-opening [148] effect of ZIF-8, where the imidazole linkers rotate to create a larger pore aperture. To confirm this, a ZIF-7/PMPS membrane was also fabricated, as ZIF-7 has the same sodalite topology as ZIF-8 and hydrophobic pores but shows little adsorption of isobutanol due to the rigid framework structure and smaller pore aperture (Figure 15). This membrane exhibited much poorer separation and permeance properties for isobutanol, which further demonstrated how ZIF-8 was responsible for the increased permeance and separation factor. The as-synthesized ZIF-8/PMPS membrane was also used to concentrate dilute solutions of ethanol, 1-propanol, 1-butanol, isobutanol and 1-pentanol. For isobutanol it was calculated that the synthesized membrane uses half the amount of energy required per unit of isobutanol when compared to a distillation process.

**ZIF-8** and **ZIF-7** have also been incorporated within PDMS membranes for pervaporative recovery of 1-butanol and acetone respectively. The ZIF-7-PDMS membrane demonstrates the highest known separation of acetone from water (1237 g·m^−2^h^−1^ and separation factor 39.1, 60 °C 1 wt % acetone) [149]. For ZIF-8, membranes were fabricated with loadings of between 1 and 5 wt %. Incorporation of ZIF-8 into the PDMS matrix increased the 1-butanol selectivity and permeability up to a loading of 2 wt %; however, at higher loadings the selectivity towards 1-butanol was reduced. This was explained by the increased chain rigidity of the PDMS, the aggregation of ZIF-8 particles at higher loadings causing defects in the membrane, and poor compatibility between ZIF-8 and the polymer [150]. This demonstrates the importance of creating a homogenously dispersed filler within the polymer matrix and the difficulty in obtaining this at high particle loading.

Fan *et al.* have since undertaken investigations into alternative methods to fabricate **ZIF-8/**PDMS membranes to prevent aggregation at higher loadings of the inorganic filler. The use of a simultaneous spray self-assembly method for fabrication of a ZIF-8-PDMS MMM was conducted [151]. A solution of ZIF-8 and PDMS and a second solution of the crosslinking agent tetraethyl orthosilicate (TEOS) and catalyst dibutylin dilaurate (DBTDL) were sprayed onto a polysulfone (PS) substrate. When compared to membranes cast from a doctor blade the sprayed membranes exhibited superior dispersion of ZIF-8 nanoparticles throughout the polymer matrices at loadings of up to 40 wt %. For a 1 wt % 1-butanol solution a ZIF-8-PDMS membrane at 40 wt % loading of ZIF-8 demonstrated a flux of 4846.2 g·m^−2^·h^−1^ and separation factor of 81.6 at 80 °C exceeding the performance of membranes produced by Bai [150] or Liu [112]. The ZIF-8-PDMS membrane was then compared to a dia(Zn)-PDMS membrane fabricated using the same procedure. The framework dia(Zn) is composed of the same building blocks as ZIF-8 (Zn and 2-methylimidazole) but it exhibits no porosity. The dia(Zn)-PDMS membranes exhibited increased flux but decreased separation factor indicating that the gaps between the polymer matrix and inorganic filler were water permselective and that the ZIF-8 nanoparticles contribute greatly to the increased permeation and separation factor exhibited by the membranes due to the selective adsorption of 1-butanol over water.

Another method employed by Fan *et al.* involved the dispersion of as-synthesised **ZIF-8** nanoparticles without drying into PDMS. A polysulfone substrate was dipped multiple times into a ZIF-8/PDMS solution before dipping in a pre-crosslinked PDMS solution to overcome any defects in the membrane caused by particle agglomeration [152]. The nanodispersed ZIF-8 membrane exhibited a high separation factor of 53 and flux of 2801 g·m^−2^·h^−1^ for separation of 5 wt % 1-butanol water at 80 °C. These examples demonstrate the importance of the fabrication method of the membrane and how it can affect the membrane performance. An optimal MMM would ideally contain a high loading of homogenously dispersed porous inorganic filler.

**Figure 18:**
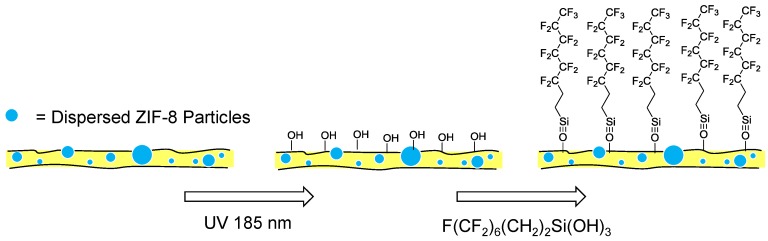
Surface modification of a ZIF-8-PDMS membrane with semi-fluorinated organosilanes as conducted in ref [153].

Further studies looking at improving the performance of ZIF-8-PDMS membranes have looked at increasing the hydrophobicity of the membrane. The surface hydrophobicity of an organophilic pervaporation membrane is closely related to the permeance of water and therefore performance of the membrane. One approach to improving the surface hydrophobicity of a ZIF-8-PDMS MMM was undertaken by Li *et al.* through the surface modification with self-assembled monolayers (SAMs) of semifluorinated (SF) organosilanes (Figure 18) [153]. The water contact angle of the ZIF-8-PDMS membrane was increased from 145.3° to 152.3° after modification with SAMs producing a superhydrophobic surface. The SAM modified ZIF-8-PDMS membrane also exhibited an increase in separation factor compared to the unmodified ZIF-8-PDMS membrane (84.8 compared to 58.4, respectively).

The improvement of the hydrophobicity of this class of membrane has also been attempted utilising **ZIF-8** incorporated into **MCM-41** particles. These MCM-41@ZIF-8 particles were then modified with a silane agent and embedded in a PDMS matrix [154]. The membranes exhibited a much greater flux of 2201 g·m^−2^·h^−1^ than the virgin membrane for separation of 5 wt % ethanol at 70 °C.

Another ZIF that has found use as an inorganic filler in MMMs is **ZIF-71**. The MOF exhibits good separation performance for aqueous mixtures of 1-propanol, 2-propanol and exhibits a greater 1-butanol separation over ZIF-8 [45]. Therefore investigations have included the use of ZIF-71 in an organophilic pervaporation membrane [155,156]. The first example was of a ZIF-71 filled PEBA MMM [155]. The inclusion of ZIF-71 into the polymer matrix increased both flux and separation factor of the membrane up to a 20 wt % loading of ZIF-71, after this separation factor was improved but flux declined. The membrane with 20 wt % loading of ZIF-71 was tested with model ABE broth solutions as well as real ABE fermentation broth. The membrane exhibited stable performance over a period of 100 hours in the pervaporation of an ABE broth with the average flux 447.9 g·m^−2^·h^−1^ and average separation factors of acetone = 8.8, 1-butanol = 18.4 and ethanol = 3.6. However, the obtained values of flux and separation factor are relatively low and have been attributed to the performance of the chosen polymer PEBA. Therefore ZIF-71 has also been incorporated into PDMS [156] due to PDMS exhibiting superior pervaporation performance for 1-butanol compared to PEBA [156].

The MOF [Zn(bdc)(ted)]_0.5_ (bdc = terephthalic acid; TED = triethylenediamine) has also been incorporated into PEBA for organophilic pervaporation of 1-butanol [157]. The framework exhibits adsorption of many organics and alcohols [158] and has been demonstrated to have good alcohol/water separation ability [38,159]. The membranes with 20 wt % loading of the MOF exhibited the greatest separation performance and were tested with a model ABE broth. The membrane had a flux of 630 g·m^−2^·h^−1^ and separation factor of 17.4 for 1-butanol at 12 g·L^−1^ within a model ABE solution at 40 °C.

The flexible MOF **MIL-53** exhibits expansion of the structure upon adsorption of methanol and ethanol [160] and has hydrophobic channels that can provide transport for ethanol molecules and reject water. Therefore it has also been incorporated into PDMS for ethanol pervaporation [161]. Loading of MIL-53 into PDMS increased the membrane affinity towards ethanol and the flux of the membrane to 5467 g·m^−2^·h^−1^ compared to the virgin PDMS membrane of 1667 g·m^−2^·h^−1^. MMMs of PDMS with non-activated MIL-53 and the starting materials for synthesis of MIL-53 (terephthalic acid and AlCl_3_·6H_2_O) were also studied. The non-activated MIL-53-PDMS membrane had a higher separation factor but much poorer flux characteristics indicating potential blockage of the pores of the MOF and the MMMs containing the starting materials exhibited performance characteristics similar to PDMS indicating that the pores of MIL-53 created preferential pathways for permeation of ethanol.

#### 5.4.3. Nanotubes, Carbons, and Other Filled MMMs for Organophilic Pervaporation

CNTs have been incorporated into organophilic pervaporation membranes for the recovery of butanol from an ABE fermentation broth [162]. CNTs were incorporated into a PEBA membrane and tested for their use for removal of 1-butanol from a 5 L fermentor. Addition of CNTs increased the removal performance of the PEBA membranes for 1-butanol. When tested with a fed-batch fermentation the mixed matrix membrane controlled the concentration of butanol at between 8 and 12 g·L^−1^ and improved the productivity and yield of the fermentation by 20%.

Another example of nanotubes for organophilic pervaporation membranes consist of polyphosphazene nanotubes (PZNTs) incorporated into PDMS for ethanol pervaporation [163]. The novel type of nanotube, PZSNTs, increased the swelling of the PDMS membranes for ethanol and successfully increased the separation factor from 5.0 to 10 for a 10 wt % ethanol solution.

**Figure 19:**
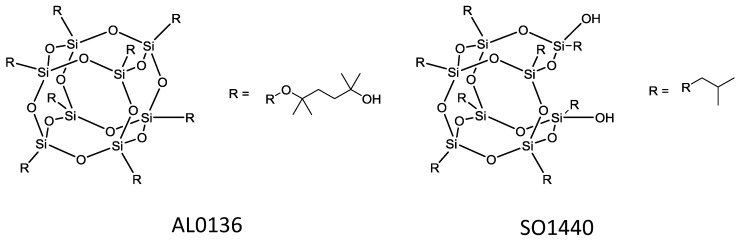
Structures of the POSSs incorporated into PEBAX as reported in [164].

The use of POSSs were also studied for creation of organophilic membranes in contrast to the work previously described in Section 5.3.3. Pebax/POSS (polyether-block-amide/polyhedral oligosilsesquioxane) MMMs were fabricated for ethanol pervaporation [164]. Two types of POSS were used to produce these membranes (Figure 19) and the optimum loading being determined at 2 wt % of POSS. At this loading, the MMM prepared with the POSS AL0136 exhibited the greater performance attributed to the POSS exhibiting a higher affinity towards ethanol.

Another important example of the incorporation of POSS into organophilic MMMs looked at tuning the free volume of a PDMS membrane to increase the permeability of 1-butanol whilst decreasing the permeability of water [165]. Initially molecular dynamics (MD) simulations were undertaken to understand the effect of addition of POSS to the packing of the polymer PDMS and these were related to experimental DSC measurements. The addition of POSS to PDMS was studied for the regulation of packing of the PDMS polymer and the effect this had on free volume of the membrane. The addition of POSS to PDMS was shown to decrease the small free volume of the PDMS membrane, which hinders the transport of smaller molecules, and an increase in the large free volume, which would aid transport of larger molecules. These findings were applied to the pervaporation of a 1 wt % aqueous butanol solution. The membranes with higher loadings of POSS exhibited greater performances in terms of selectivity and butanol permeability due to the increase in the large free volume of the polymer membrane aiding 1-butanol permeation and the decrease in small free volume hindering permeation of water.

#### 5.4.4. Implications: MMMs for Organophilic Pervaporation

Overall these studies have shown that inorganic fillers have been hugely influential on improving the performance characteristics of organophilic pervaporation membranes. Although organophilic mixed matrix pervaporation membranes have not been applied commercially to the recovery of fermentation products, it is clear that they are one of the leading low energy technologies within this field of research.

Future opportunities for the use of MMMs in organophilic pervaporation include:

- Further studies into the fouling and long term stability of high-performing MMMs for organophilic pervaporation are required to help develop measures to overcome fouling from the many varying components of microbial fermentation broths.
- Application of the varying membrane fabrication techniques developed for ZIF-8-PDMS MMMs to other MOFs and other inorganic fillers.
- Bespoke polymer development to complement and enhance the different fillers and separations that the resulting MMMs will be applied to.
- Full cost-benefit analysis of the use of novel, expensive, inorganic fillers within these MMMs investigating whether the improved membrane performance characteristics can offset the increased membrane fabrication costs.

## 6. Fabrication methods for MMMs

As described above, there is a huge library of polymers and inorganic materials that have been utilized within MMMs applicable to the separation of fermentation broths. It is clear that both the inorganic material and chosen polymer can have a large effect on both the permeability and separation ability of the membrane. However, as is evident in several of the examples, the fabrication method of the MMMs can also ultimately have a large influence on the ultimate performance of the membrane.

### 6.1. Fabrication methods for TFN Membranes for NF and RO

**Figure 20:**
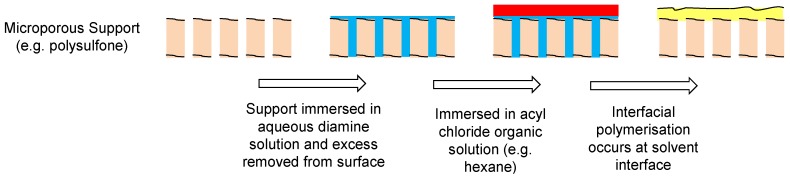
Schematic of interfacial polymerization.

**Figure 21:**
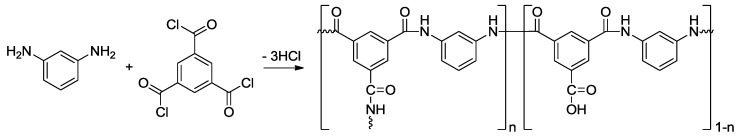
Interfacial polymerization between 1,3-phenylenediamine and trimesoyl chloride to produce a fully aromatic polyamide.

The most common procedure for forming TFC polymeric membranes for NF and RO is through interfacial polymerization [55,169]. The two monomers are dispersed in two immiscible solvents (e.g., water and hexane) and the polymerization reaction occurs at the interface. For example, the polyamide layer of a TFC membrane is produced by immersing a microporous support (e.g., polysulfone) in an aqueous solution of the diamine (e.g., 1,3-phenylenediamine). The excess solution is removed from the support and put in contact with an organic solution containing an acyl chloride (e.g., trimesoyl chloride in hexane). The polyamide is then formed on the surface of the microporous support (Figure 20 and Figure 21).

To incorporate an inorganic nanomaterial into the polyamide matrix through interfacial polymerization, the nanomaterial is generally dispersed in either the aqueous or organic solution during fabrication. Problems can arise though during fabrication in this manner:

- Agglomeration of the inorganic phase within the polyamide layer can reduce both the surface area of the particles and create non-selective voids within the polyamide layer. This is due to the poor dispersion of the inorganic nanoparticles in either solution used for the interfacial polymerization.
- Leaching of the inorganic component out of the membrane can occur due to a lack of compatibility or chemical bonding between the polymer and inorganic phase.

Fabrication challenges in TFN for NF and RO has been reviewed in detail recently by Lau *et al.* [63]. A number of examples are presented attempting to increase the dispersion of nanoparticles in the interfacial polymerization solutions, as well as utilizing novel interfacial polymerization methods to overcome the problems raised above. As this class of membrane has yet to be applied to the purification/concentration of a fermentation broth, it has therefore not been discussed in further detail here. This, however, provides a vast area of research to be investigated with concerns arising especially from potential leaching of the inorganic material out of the membrane. For example, if a recycle is used to return the purified liquid back to the fermenter, potentially hazardous inorganic nanoparticles could contaminate and cause problems with the fermentation due to toxicity towards the microorganisms. Future research should focus on this extremely important issue.

### 6.2. Fabrication Techniques of MMMs for Pervaporation

The majority of MMMs for pervaporation presented above are produced through the creation of a polymer dope solution and solution casting by either pouring on a glass plate or with a doctor blade [149,150,155,156,157]. Other fabrication methods include dip coating [112,152] and simultaneous spray self-assembly [151]. The main challenges in fabrication of MMMs for pervaporation are similar to those for TFN membranes as presented above:

- Agglomeration of inorganic particles reducing available surface area and potentially creating non-selective voids;
- Incompatibility between the inorganic phase and polymer matrix creating non-selective voids;
- Leaching of the inorganic phase out of the polymer matrix.

An important procedure in each of these examples is the dispersion of the inorganic particles within the polymer dope solution. Each example uses a combination of stirring and sonication in an attempt to increase the dispersion of the inorganic phase. For their ZIF-8-PMPS membranes Liu *et al.* [112] noted several ways to improve the dispersion of ZIF-8 within the membrane: the use of newly synthesized ZIF-8 nanoparticles, dispersion of the nanoparticles in isooctane before mixing with the polymer, and utilizing a probe type sonicator rather than an ultrasonic bath.

As described previously (Section 5.4.2) Fan *et al.* [151] developed a novel method for improving the dispersion of ZIF-8 within a PDMS membrane called simultaneous-spray self-assembly. The membranes produced have exhibited some of the highest performances for organophilic pervaporation of 1-butanol and this has been attributed to the increased dispersity of the inorganic filler and homogeneity of the membrane. This demonstrates that development of novel fabrication techniques can produce MMMs with superior performance characteristics and will become an important direction for research into MMMs for pervaporation in future.

To overcome the incompatibility between the polymer and inorganic phases of the MMMs, many examples also primed the inorganic particles through surface modification. One of the most common examples presented include silylation of the surface of the inorganic particles [134,136,137,138,154]. The incorporation of silyl groups onto the surface of the inorganic phases is thought to improve the compatibility with PDMS. Obviously though the choice of surface modification of the inorganic particle must be compatible with the chosen polymer. Therefore for the hydrophilic MMM of ZIF-90 and P84 polyimide [108] (Section 5.3.2) the ZIF-90 nanoparticles were primed with a sulfonated poly(ether sulfone) surface modification. The effect of the SPES modification is thought to increase particle dispersion through preventing agglomeration, improve affinity between polymer and nanoparticle, and, increase the hydrophilicity of the membrane. Generally an improvement in the membrane performance is observed for these types of modifications, proving that this could be an important step in fabricating MMMs with even greater separation and permeability characteristics.

The particle size of the inorganic phase in a MMM is also important. Smaller nanosized particles if able to be dispersed well in the casting solutions would create a more homogenous matrix and create less void space. One example is the use of nanosized silicalite-1 in PDMS [134]. The study found that membranes incorporated with nano-sized silicalite-1 always had a higher total flux and separation factor than those membranes fabricated from micron sized silicalite-1 for pervaporation of a 6 wt % ethanol solution. As the library of available nanosized materials increases and the synthetic procedures for producing these materials simplify [170] many more examples of including these nanosized materials in MMMs for pervaporation should be sought.

For the same reasons as for TFN membranes, leaching of the inorganic phase out of the polymer membrane would also be problematic for pervaporation membranes. Searching the literature, however, has found little evidence of research into leaching of nanoparticles out of fabricated MMMs for pervaporation. This is incredibly important and should be an area of future research in this field.

## 7. Other Examples of MMMs for Fermentative Separation Applications

**Figure 22:**
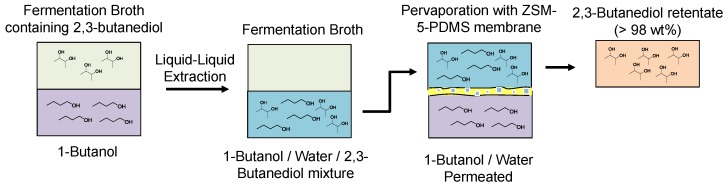
Schematic of the recovery process for 2,3-butanediol studied in [172].

Some fermentation products, however, do not lend themselves towards direct hydrophilic or organophilic pervaporation. For example 2,3-butanediol exhibits relatively high hydrophilicity due to the two hydroxyl groups present as well as a high boiling point and low vapour pressure, meaning it is difficult to find a material that can preferentially permeate 2,3-butanediol over water. Therefore a recovery method utilizing solvent extraction then pervaporation was designed (Figure 22) [171]. 2,3-butanediol was initially recovered from a fermentation broth using 1-butanol as extracting solvent and then 2,3-butanediol was concentrated in the retentate by removing water and 1-butanol with a PDMS pervaporation membrane to purities in excess of 98 wt %. The process was further improved through the use of a ZSM-5 filled PDMS membrane for removal of water and 1-butanol from the extracted phase [172].

This work demonstrates that membranes and MMMs have a role to play in the purification and fractionation of the products from fermentations. However, this may not be as the sole unit operation but as part of an integrated separation process, incorporating a number of complimentary and synergistic separation unit operations. Membranes, and especially MMMs, can be used to improve other extraction processes (such as liquid-liquid extraction as described above), if this is the most effective, low-cost and efficient route to the separation. Other examples of hybrid membrane processes that have been used in related separations include distillation/pervaporation [173] and membrane assisted vapour stripping (MAVS) [174]. MMMs could provide improved performance of the membranes utilized in these processes. A complete process integration and intensification study should be done to explore such options in order to determine the true optimal process configuration for the recovery of fermentation products.

## 8. Conclusions

This review article has presented a number of examples of MMMs which are relevant to the separation and recovery of fermentation products. For the application of membranes to fermentative separations, novel membranes that can provide greater separation and flux performances are required for commercial application. It is clear from the literature presented in this review that MMMs are currently providing some of the most high-performing membranes for these separations, but a number of problems for the fabrication of MMMs has been noted:

- The intrinsic property of the inorganic filler to be used should be fully understood when utilizing it for a specific MMM separation; novel inorganic materials that have shown exemplary separation performance should be studied.
- The compatibility between the polymer and inorganic filler is important to prevent the formation of defects between the polymer and inorganic components and creating non-selective voids.
- Agglomeration of the inorganic filler should be limited, allowing for high loadings of homogeneously dispersed filler within the polymer matrix.
- Potential for leaching of the inorganic phase out of the polymer membrane.

A number of opportunities for future research and development into the use MMMs for fermentation separations have also been identified for each of the different membrane classes relevant to fermentation separations (*i.e.*, pressure filtrations and pervaporations), and the overall opportunities are as follows:

For pressure filtrations (UF, NF, RO):

- There has been little work in the use of MMMs to improve flux and selectivity in UF, NF and RO membranes for the separation of fermentation products, so this is a significant area for future research with great opportunities in investigating a wide range of inorganic fillers, polymers and fermentation applications.

For pervaporations (hydrophilic and organophilic):

- Further investigations into the effect of different membrane fabrication techniques on the performance of MMMs for applicable separations to fermentations.
- Further screening of a range of different water stable MOFs as inorganic fillers.
- Further studies into the fouling and long-term stability of high-performing MMMs to help develop measures to overcome fouling from the many varying components of microbial fermentation broths.

For both membrane process classes:

- Bespoke polymer development to complement and enhance the different fillers and separations that the resulting MMMs will be applied to.
- Improved fabrication and operational methods for preventing the leaching of the inorganic phase out of the polymer membrane.
- Full cost-benefit analysis of the use of novel, expensive, inorganic fillers within these investigated MMMs is needed to determine whether the improved membrane performance characteristics can offset the increased membrane fabrication costs.

Overall it is clear that the development of new processes for the recovery of dilute fermentation products is important, since these processes are the reactor work-horses in the replacement of petrochemicals with chemicals from renewable sources. Since the removal of water is therefore key in the separation and purification of the products from fermentations and related processes, and membrane processes offer a low energy-alternative, membrane processes and MMMs will play a significant role in the development and proliferation of these fossil fuel replacement processes.

## Figures and Tables

**Table 1:** Application of different membrane processes for separations within microbial fermentation.

Driving Force	Membrane Process	Pore Size/Molecular Weight Cut-Off (MWCO)	Separation of:	Literature:
Pressure	Microfiltration	0.1–10 μm	Yeast, Bacteria	[9,10,11]
	Ultrafiltration	1–100 nm or >2000 g·mol^−1^	Proteins, DNA	[12]
	Nanofiltration	200–2000 g·mol^−1^	Sugars, Acids, Salts	[13,14,15]
	Reverse Osmosis	<100 g·mol^−1^	Alcohols, Acids, Salts	[16,17]
Vapour Pressure	Pervaporation/Vapour Permeation	–	Water or Organic Solvent	[18]

**Table 2:** Hydrophilic pervaporation of ethanol-water mixtures by various polymer membranes.

Dehydration of: Alcohol/Water (wt %/wt %)	Polymer	Inorganic Filler	Membrane Performance		Temperature (°C)	Membrane Thickness (μm)	Ref
			Total Flux (kg·m^−2^·h^−1^)	Separation Factor (α)			
Ethanol/Water (90/10)	Polyacrylonitrile	–	0.03	12500	70	50	[78]
Ethanol/Water (90/10)	Polyacrylamide	–	0.42	2200	70	50	[78]
Ethanol/Water (90/10)	Polyvinylalcohol	–	0.38	140	70	50	[78]
Ethanol/Water (90/10)	Polyethersulfone	–	0.72	52	70	50	[78]
Ethanol/Water (80/20)	Polyvinylalcohol	–	0.14	40	50	70–80	[83]
Ethanol/Water (80/20)	Polyvinylalcohol	Zeolite NaX (11 wt %)	0.21	19.4	50	70–80	[83]

**Table 3:** Organophilic pervaporation by various polymer membranes.

Organophilic Pervaporation of:	Polymer	Membrane Performance		Temperature (°C)	Membrane Thickness (μm)	Ref
		Total Flux (kg·m^−2^·h^−1^)	Separation Factor (α)			
1 wt % Ethanol	PTMSP	–	10.7	75	100	[87]
2 wt % Ethanol	PTFE (0.2 μm pore diameter)	12	2	60	175	[88]
5 wt % Ethanol	PTFE (0.2 μm pore diameter)	4	8	30	80	[89]
1 wt % Ethanol	PIM-1	0.47	10.7	30	25–40	[90]
5 wt % Ethanol	Poly(octylmethyl siloxane) POMS	0.12	3.95	50	–	[91]
5 wt % Ethanol	PEBA 2533	0.37	2.4	23	100	[92]
1.5 wt % Butanol	PDMS	0.72	33.7	55	30	[93]
5 wt % Butanol	PEBA 2533	0.65	8.2	23	100	[92]

**Table 4:** Examples of MMMs for hydrophilic pervaporation.

Dehydration of: Alcohol/Water (wt %/wt %)	Inorganic Filler	Polymer	Neat Membrane Performance		MMM Performance		Temperature (°C)	Ref
			Total Flux (g·m^−2^·h^−1^)	Separation Factor (α)	Total Flux (g·m^−2^·h^−1^)	Separation Factor (α)		
Ethanol/Water (90/10)	H-ZSM-5 (8 wt %)	Chitosan	54.18	158	231	153	80	[102]
Ethanol/Water (90/10)	MPTMS-modified H-ZSM-5 (8 wt %)	Chitosan	120	175	278	274	80	[103]
Ethanol/Water (90/10)	Functionalized-MWCNT (2 wt %)	Chitosan	112	580	337	570	30	[121]
Ethanol/Water (80/20)	Zeolite KA (11 wt %)	PVA	140	40	164	40	50	[83]
Ethanol/Water (80/20)	Zeolite NaA (11 wt %)	PVA	140	40	172	36.6	50	[83]
Ethanol/Water (80/20)	Zeolite CaA (11 wt %)	PVA	140	40	194	22.3	50	[83]
Ethanol/Water (80/20)	Zeolite NaX (11 wt %)	PVA	140	40	214	19.4	50	[83]
Ethanol/Water (85/15)	ZIF-8 33.7 wt %	PBI	151	4	106	25.4	60	[105]
Ethanol/Water (85/15)	ZIF-8 58.7 wt %	PBI	151	4	992	10	60	[105]
Ethanol/Water (90/10)	ZIF-7 (5 wt %)	Chitosan	602	148	322	2812	25	[110]
Ethanol/Water (90/10)	ZIF-8 (12 wt %)	Matrimid 5218 (polyimide)	240	260	260	300	42	[107]
Ethanol/Water (90/10)	MCM-41 3.1 μm (12 wt %)	Matrimid 5218 (polyimide)	240	260	310	190	42	[107]
Ethanol/Water (90/10)	MCM-41 0.53 μm (12 wt %)	Matrimid 5218 (polyimide)	240	260	440	252	42	[107]
Ethanol/Water (90/10)	MCM-41-ZIF-8 coated (12 wt %)	Matrimid 5218 (polyimide)	240	260	200	137	42	[107]
Ethanol/Water (85/15)	POSS (AMO273) (2 wt %)	6FDA-NDA/DABA	–	–	1900	166	60	[119]
Ethanol/Water (85/15)	POSS (SO1440)	6FDA-NDA/DABA (3 wt % sulfonated polyimide)	–	–	2000	237	60	[120]
IPA/Water (90/10)	Na^+^MMT (5 wt %)	PVA	95	77	51	1116	30	[118]
IPA/Water (90/10)	Na^+^MMT (10 wt %)	PVA	95	77	75	2241	30	[118]
IPA/Water (90/10)	Silicalite-1 (20 wt %)	PVA	95	77	69	2241	30	[122]
IPA/Water (87.7/13.3)	Aluminosilicate (6 wt %)	PVA-glutaraldehyde crosslinked			109.8	73	40	[100]
IPA/Water (90/10)	ZSM-5 (6 wt %)	PVA	–	–	320	216	30	[123]
IPA/Water (80/20)	Zeolite KA (11 wt %)	PVA	146	223	179	410	50	[83]
IPA/Water (80/20)	Zeolite NaA (11 wt %)	PVA	146	223	183	328	50	[83]
IPA/Water (80/20)	Zeolite CaA (11 wt %)	PVA	146	223	190	233	50	[83]
IPA/Water (80/20)	Zeolite NaX (11 wt %)	PVA	146	223	216	233	50	[83]
IPA/Water (90/10)	Zeolite 5A (20 wt %)	P84	30	3000	40	4200	60	[97]
IPA/Water (90/10)	Zeolite 13X (40 wt %)	P84	30	3000	110	2700	60	[97]
IPA/Water (90/10)	Zeolite 4A (10 wt %)	Matrimid 5218	14	12716	18	8991	30	[99]
IPA/Water (90/10)	ZSM-5 (10 wt %)	Matrimid 5218	14	12716	16	3904	30	[99]
IPA/Water (95/5)	NaY (30 wt %)	Chitosan	32	422	115	2620	30	[124]
IPA/Water (87.4/12.6)	Zeolite-K-LTL (10 wt %)	Sodium Alginate	–	–	140	3847	30	[96]
IPA/Water (90/10)	ZIF-8 (5 wt %)	PVA	135	163	868	132	30	[104]
IPA/Water (90/10)	ZIF-8 (7.5 wt %)	PVA	135	163	952	91	30	[104]
IPA/Water (85/15)	ZIF-8 (33.7 wt %)	PBI	13	>5000	103	1686	60	[105]
IPA/Water (85/15)	ZIF-8 (58.7 wt %)	PBI	13	>5000	246	310	60	[105]
IPA/Water (85/15)	ZIF-90 (30 wt %)	P84	–	–	114	385	60	[108]
IPA/Water (85/15)	ZIF-90-SPES (30 wt %)	P84	–	–	109	5668	60	
IPA/Water (90/10)	CNTs 1 wt %	PVA	–	–	96	817	30	[113]
IPA/Water (90/10)	CNTs 2 wt %	PVA	–	–	79	1794	30	[113]
IPA/Water (90/10)	MWNT-PAH (1 wt %)	PVA	229	141	207	945	30	[114]
IPA/Water (90/10)	HPA 40–50 μm (7 wt %)	PVA	132	77	32	89991	30	[117]
n-Butanol/Water (85/15)	ZIF-8 (33.7 wt %)	PBI	11.6	>5000	81	3417	60	[105]
n-Butanol/Water (85/15)	ZIF-8 (58.7 wt %)	PBI	11.6	>5000	226	698	60	[105]

**Table 5:** Examples of MMMs for hydrophobic pervaporation.

Organophilic Pervaporation of:	Inorganic Filler	Polymer	Neat Membrane Performance		MMM Performance		Temperature	Ref
			Total Flux (g·m^−2^·h^−1^)	Separation Factor (α)	Total Flux (g·m^−2^·h^−1^)	Separation Factor (α)		
5 wt % Methanol	[Cu^II^_2_(bza)_4_(pyz)]_n_ (3 wt %)	PDMS	24	2	33	6.5	RT	[147]
5 wt % Ethanol	ZSM-5 (40 wt %)	PDMS	–	–	408	14	40	[166]
5 wt % Ethanol	ZSM-5 HF etched	PDMS	–	–	211	9.2	50	[167]
5 wt % Ethanol	Silicalite-1 (60 wt %)	PDMS	24	7.6	50.7	16.5	22.5	[128]
5.1 wt % Ethanol	Silicalite-1 (77 wt %) TFC	PDMS	530	4.4 (7.0 wt % EtOH Feed)	150	34	22	[129]
1.6 wt % Ethanol	Silicalite-1-VTMS	PDMS	–	–	–	18	50	[137]
5 wt % Ethanol	Silicalite-1 (2 wt %)	PEBA	–	–	833	3.6	40	[139]
1 wt % Ethanol	-	PIM-1	470	10.7	–	–	30	[90]
5 wt % Ethanol	Silicalite-1 (19.3 wt %)	PIM-1	6520	3.61	5460	5.68	60	[143]
5 wt % Ethanol	Silicalite-1 (2 wt %)	PEBA	–	–	833	3.6	40	[139]
5 wt % Ethanol	MIL-53 (40 wt %)	PDMS	1667	7.6	5467	11.1	70	[161]
5 wt % Ethanol	MCM-41@ZIF-8	PDMS	886	6.8	1846	9.5	60	[154]
5 wt % Ethanol	[Cu^II^_2_(bza)_4_(pyz)]_n_ (3 wt %)	PDMS	23	2.3	47	6.2	RT	[147]
10 wt % Ethanol	PZSNTs (10 wt %)	PDMS	–	–	11.9 × 10^−3^ g·mm^−2^·h^−1^	10	40	[163]
5 wt % Ethanol	POSS (AL0136) (2 wt %)	PEBAX 2533	–	–	183.5	4.6	RT	[164]
5 wt % Ethanol	POSS (SO1440) (2 wt %)	PEBAX 2533	–	–	125.8	4.1	RT	[164]
1% 1-butanol	Silicalite-1	PDMS	–	–	607	93	70	[132]
4.3 wt % 1-butanol	ZSM-5 (5 wt %)	PEBA	–	–	719.3	33.3	35	[146]
2.5 wt % 1-butanol	MCM-41 (2 wt %)	PEBA	–	–	> 500	25	35	[168]
1.0 wt % isobutanol	ZIF-8 (10 wt %)	PMPS	–	–	6400	40.1	80	[112]
1 wt % 1-butanol	ZIF-8	PMPS	–	–	5100	36.8	80	[112]
0.96 wt % 1-butanol	ZIF-8	PDMS	2.59 (Permeability of n-butanol × 10^5^ barrer)	3.21	1.71 (Permeability of n-butanol × 10^5^ barrer)	5.95	40	[150]
5 wt % 1-butanol	ZIF-8 (nanodisperse)	PDMS	–	–	2800.5	52.8	80	[152]
1 wt % 1-butanol	ZIF-8 (40 wt %) Simultaneous spray self-assembly	PDMS	–	–	4846.2	81.6	80	[151]
3 wt % 1-butanol	ZIF-8	PDMS	1065	13.4	1459	58.4	60	[153]
3 wt % 1-butanol	ZIF-8	PDMS^CF3^	1049	19.4	1339	84.8	60	
3 wt % 1-butanol	MCM-41@ZIF-8	PDMS	–	–	2052	45	60	[154]
Model ABE Broth / 1-Butanol (12 g/L)	ZIF-71	PEBA	–	–	520	18.8	37	[155]
Model ABE Broth 1-Butanol (12 g/L)	Zn(BDC)(TED)_0.5_	PEBA	–	–	630	17.4	40	[157]
1 wt % 1-butanol	CNT 5 wt %	PEBA	85	17.4	153	19.4	37	[162]
1 wt % 1-butanol	CNT 10 wt %	PEBA	85	17.4	139	18	37	[162]
1 wt % 1-butanol	POSS	PDMS	–	–	745	40	40	[165]
Acetone	ZIF-7	PDMS	–	–	1236.8	39.1	60	[149]
